# Developmental Milestones in Children’s Environmental Health

**DOI:** 10.1289/ehp.1002957

**Published:** 2010-10

**Authors:** Ruth A. Etzel

**Affiliations:** Senior Officer for Environmental Health Research, Department of Public Health and Environment, World Health Organization, Geneva, Switzerland, E-mail: ETZELR@WHO.INT

This month the children’s environmental health (CEH) movement reaches the age of 21 years, an important milestone. The movement began in California in October 1989 with the launch of The Kids in the Environment Project, designed to train health professionals about environmental health and children. This project subsequently evolved into the Children’s Environmental Health Network (http://www.cehn.org/)*,* a national organization headquartered in Washington, DC. Pediatricians’ interest in children’s unique susceptibility to environmental hazards probably dates further back, at least to 1954, when fallout from a nuclear weapons test on Bikini Island in the South Pacific caused acute radiation burns among people living on neighboring islands. Two boys exposed to fallout when they were infants developed severe hypothyroidism and short stature in mid-childhood. Young children were more severely affected than adults; among 35 children < 15 years of age, 3 developed carcinoma of the thyroid, compared with 2 among 46 persons who were ≥ 15 years of age at exposure (Merke and Miller 1992). These cases highlighted children’s special vulnerability to ionizing radiation. Because of concerns about fallout from weapons testing and fears of nuclear war, in 1957 the American Academy of Pediatrics established what is now known as the Committee on Environmental Health to address these issues. For 53 years this committee has been the world leader in advocacy and education about risks to children from environmental hazards, publishing the first book on pediatric environmental health in 1999 (Etzel 1999).

Despite these activities, modern medical and nursing school curricula in the United States have been slow to include information about the environment’s impact on child health. In the early 1900s, the environment was already well understood to be an important contributor to health, and its importance was routinely taught to students. Think of Florence Nightingale’s 6 Ds of disease: dirt, drink (clean drinking water), diet, damp, drafts, and drains (proper drainage and sewage systems). With the advent of high-tech medicine, however, these fundamentals began to receive short shrift in graduate education. At the same time, the house call—a home visit that allowed the physician to view the environment in which the patient lived—became increasingly rare, and doctors’ visits began to take place in the confines of modern offices or hospitals, far removed from the day-to-day surroundings of the family. Over time, the environment became “invisible” to the medical practitioner.

To counteract this phenomenon, the CEH movement advocated for the development of specific research programs and training programs to introduce nursing and medical students to the concepts of children’s vulnerability to environmental hazards. The CEH movement also encouraged academic and health organizations to study the impact of the environment on infant and child health. As a result, beginning in 1998, numerous Centers for Children’s Environmental Health and Disease Prevention Research, funded by the National Institute of Environmental Health Sciences, were established in U.S. universities to study and prevent a range of childhood diseases and outcomes that can result from environmental exposures.

Nonetheless, in the early 2000s, physicians in the United States who were interested in careers in pediatric environmental health could find no formal fellowship training programs. In 2002, through the Academic Pediatric Association, the first formal fellowship training programs in pediatric environmental health were initiated. These 3-year training programs are designed to provide pediatricians with specific competencies to enable them to undertake environmental health research, teaching, and advocacy (Etzel et al. 2003). Fellowship training is available in many large U.S. cities including Boston, Massachusetts; New York, New York; Cincinnati, Ohio; Pittsburgh, Pennsylvania; San Francisco, California; and Seattle, Washington, as well as Vancouver, Canada.

Although the CEH movement began in the United States, within a decade it had become an international movement. In 1999 the World Health Organization (WHO) set up a Task Force for the Protection of Children’s Environmental Health and began developing training materials on children’s health and the environment for an international audience (Pronczuk de Garbino 2005; United Nations Environment Programme, United Nations Children’s Fund, WHO 2002), including a Training Package for Health Care Providers, a set of peer-reviewed slide sets covering the major environmental issues for children (WHO 2010c). These slide sets (available free from WHO) have been used extensively to teach practicing physicians about pediatric environmental health issues not usually covered in the traditional medical school or postgraduate curriculum. In 2005, the International Pediatric Association, in collaboration with the WHO, launched the International Pediatric Environmental Health Leadership Institute to provide special training for doctors and nurses on children’s health and the environment using the WHO Training Package for Health Care Providers (WHO 2010c). After attending the training course, participants who completed a community project, gave a presentation at their home hospital, and documented children’s environmental diseases in their medical records were offered the opportunity to sit for an examination toward a special certificate in Children’s Environmental Health. Diplomates of the Institute now provide training on CEH in many countries.

In addition to educating physicians, the WHO aimed to raise policy makers’ awareness about children’s health and the environment. To that end, the WHO has organized three large international conferences for this audience. In 2002 the 1st WHO International Conference on Environmental Threats to the Health of Children was held in Bangkok, Thailand. The conference focused on science-oriented issues, research needs, and capacity building while addressing the concrete needs for action and policies at the community, country, regional, and international levels. The 2nd WHO International Conference on Children’s Environmental Health was held in Buenos Aires, Argentina, in 2005. The 3rd WHO International Conference on Children’s Health and the Environment was held in Busan, Republic of Korea, in 2009, resulting in a Global Plan of Action for Children’s Health and the Environment (WHO 2010a).

This issue of *Environmental Health Perspectives* documents that substantial progress has been made in research to understand the role of the environment in the illnesses of childhood and adolescence. Consideration of illnesses traditionally associated with the environment, such as waterborne and foodborne diseases, has expanded to include study of natural toxins and toxic chemicals that derive from the rapid expansion of industry and technology. But much remains to be done to ensure that our burgeoning knowledge is translated into preventive actions for children.

Why has it been so difficult to move from knowing to doing? First, many of the decisions affecting children are made not by those in the health sector, but by our professional colleagues in the agriculture, education, energy, housing, mining, and transportation sectors. Just as “men are from Mars and women are from Venus,” it seems as if professionals in each of these sectors are from different planets. Although we may speak the same language, we rarely have more than a cursory understanding of the forces that shape one another’s decisions and other considerations. Because the CEH movement has focused on educating the health community, few efforts have been made to establish relationships with other economic sectors. To use a developmental analogy, we are still involved primarily in “parallel play” rather than team sports. Professionals in the health sciences may work alongside professionals in other sectors, but we are absorbed in our own activities and usually have little interaction outside them. Instead of sitting at the table with urban planners, housing specialists, and energy experts when health professionals are planning an approach to a child health problem such as asthma, we usually move forward to design a study, implement it, analyze the results, and then present it as a fait accompli to our colleagues in other economic sectors, and hope that they will find it useful.

This is not the ideal way to engage them. We medical professionals need to fully engage with other sectors as we launch our attempts to find solutions to child health problems. Major breakthroughs are likely to occur in protecting children from hazards in the environment only when we establish strong working relationships with those who haven’t been trained as we have and who don’t think as we do. One tool that helps different sectors to interact is Health Impact Assessment, promoted by the WHO (2010b) and by many countries including the United States. Health Impact Assessment helps decision makers make choices about alternatives and improvements to prevent disease/injury and to actively promote health. A recent White House Task Force on Childhood Obesity report recommends that communities consider integrating Health Impact Assessment into local decision-making processes before undertaking any major new development or planning initiative (White House Task Force on Childhood Obesity 2010).

Although pediatric environmental health, now 21 years old, can sit at the same table with other recognized pediatric subspecialities, and although some of the developmental milestones have been achieved, we have a ways to go in turning our considerable knowledge into action. Because the risk factors for environmentally related diseases reside in sectors beyond the direct control of public health, “parallel play” activities should give way to fully cooperative endeavors with other sectors.

## Figures and Tables

**Figure:**
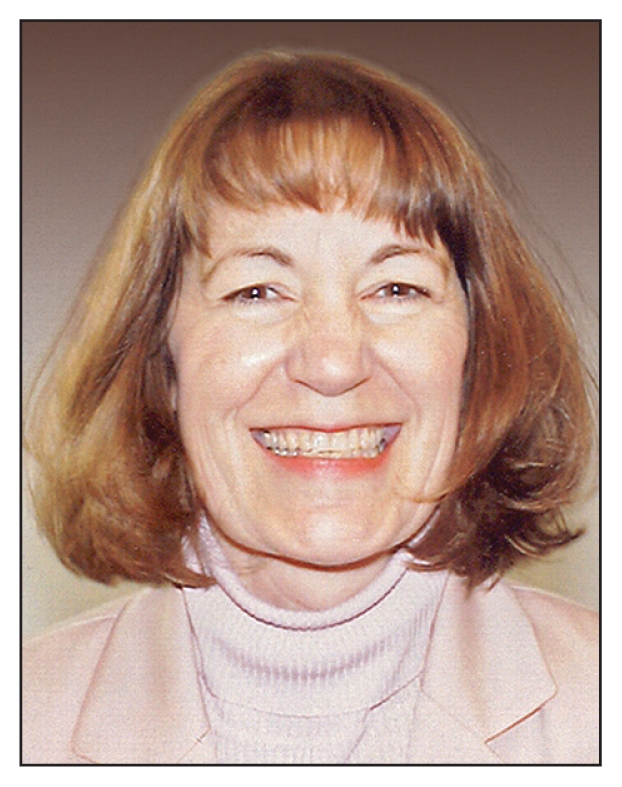
Ruth A. Etzel
